# Development and validation of a Weight Literacy Scale in English and Spanish

**DOI:** 10.1371/journal.pone.0204678

**Published:** 2018-10-24

**Authors:** Monica L. Wang, Tariana V. Little, Christine Frisard, Amy Borg, Stephenie C. Lemon, Milagros C. Rosal

**Affiliations:** 1 Department of Community Health Sciences, Boston University School of Public Health, Boston, Massachusetts, United States of America; 2 Department of Social and Behavioral Sciences, Harvard T.H. Chan School of Public Health, Boston, Massachusetts, United States of America; 3 Division of Preventive and Behavioral Medicine, University of Massachusetts Medical School, Worcester, Massachusetts, United States of America; University of Auckland, NEW ZEALAND

## Abstract

**Objectives:**

To develop and validate a Weight Literacy Scale in English and Spanish for adults.

**Methods:**

The two-phase study utilized quantitative and qualitative methods. Phase 1 of the study consisted of developing an initial survey (English and Spanish versions) assessing weight literacy based on a review of the literature; conducting semi-structured interviews with content experts (N = 9) to refine survey items; and conducting in-person cognitive interviews with 20 study participants (N = 10 English-speaking and N = 10 Spanish-speaking adults) for survey pre-testing. Survey items were modified based on Phase 1 findings. Phase 2 consisted of a psychometric study of the Weight Literacy Scale developed in Phase 1. Procedures included administering the Weight Literacy Scale to 200 study participants (N = 100 English-speaking and N = 100 Spanish-speaking adults), a quantitative survey assessing dietary and physical activity behaviors and sociodemographics, measuring participants’ height and weight, and assessing the scale’s validity and internal reliability. A subset of Phase 2 participants (N = 71) completed the weight literacy scale at two-weeks follow-up to assess test-retest reliability. Participant recruitment and study procedures took place in community settings in central Massachusetts for both study phases. Weight literacy scale scores were calculated as the sum of total correct items. Three rounds of factor analysis were performed to identify items for elimination. The Kuder Richardson’s Coefficient of reliability was calculated. Correlations between the Weight Literacy Scale scores and related measures (body mass index and weight status, dietary behaviors, physical activity behaviors, and confidence in filling out medical forms) were examined.

**Results:**

The final scale included 31 items and demonstrated strong internal consistency (Kuder Richardson Coefficient = 0.90), reasonable construct validity, and acceptable test-retest reliability (ρ = 0.72).

**Conclusion:**

The Weight Literacy Scale is a reliable and valid research instrument to assess weight literacy among English- and Spanish-speaking adults.

## Introduction

Obesity is a growing epidemic nationally and globally [1], with over one third (36.5%) of U.S. adults being currently obese [2, 3]. Given the premature morbidity and mortality and staggering health care costs associated with obesity [4], interventions that successfully help adults prevent and manage obesity through behavior change (e.g., diet, physical activity) are needed.

Several factors facilitate successful weight management, including health literacy, defined by the Institute of Medicine as "the degree to which individuals have the capacity to obtain, process, and understand basic health information and services needed to make appropriate health decisions [5]." Studies suggest an inverse association between health literacy and body mass index (BMI), with lower health literacy associated with higher BMI among adults [6, 7]. Studies examining health literacy within the context of obesity and/or weight loss efforts have primarily focused on assessing participants’ sources of health information. An increasing percentage of individuals, particularly those who are obese, utilize the internet and mobile apps as the primary sources of weight loss information and advice [8, 9], though some research has found that overweight or obese African American women reported television shows (e.g., Oprah, The Biggest Loser) as the main source of dieting information [10]. Other common sources of weight loss information include in-person exchanges with friends/family, publications like women’s magazines, diet books and newspapers, and health personnel i.e. physicians, nurses, and dieticians [8, 10, 11].

Health literacy may play an important role in facilitating successful behavioral weight loss or management, though the mechanisms of this association are not well established. One study among obese African American women found that participants with average health literacy levels were more likely to join weight loss programs, increase physical activity for weight loss, and seek weight loss information online compared to participants with low health literacy levels [11]. Among overweight and obese adults in a behavioral weight loss intervention, high health literacy was significantly correlated with successful weight loss [12]. Even less research has been conducted on the assessment of health literacy specific to weight loss or management. Health literacy in the context of weight management is complex and requires an in-depth understanding of the knowledge, skills, and behavioral targets needed to make informed decisions about weight management. Understanding individuals’ capability to obtain and process weight loss information and make decisions regarding weight management, or “weight literacy,” may help elucidate barriers and facilitators of successful weight loss.

Building on the definition of health literacy from the Institute of Medicine, we propose to define weight literacy as “the degree to which individuals can obtain, process, understand, and communicate about weight-related information needed to make informed decisions about weight management.” This definition reflects our view for the need for health care consumers to be able to communicate their understanding of weight-related information; substituted “basic” and “health” information for weight-related information specifically; and focused on “informed” decision-making because “appropriate” care may not be culturally congruent with the patient and an “informed” decision is more patient-centered. We distinguish weight literacy from nutrition literacy, defined as “the degree to which individuals have the capacity to obtain, process, and understand *nutrition* information and skills needed in order to make appropriate *nutrition* decisions” [13], as weight literacy encompasses information and skills specific for weight loss or management (e.g., energy balance, goal-setting and tracking for diet and physical activity).

To our knowledge, a validated weight literacy scale that is linguistically appropriate for ethnically diverse populations does not exist. Such a scale would be instrumental in enhancing our understanding of the role of weight literacy in behavioral weight loss/management and identifying areas to improve weight literacy and ultimately weight loss/management behaviors. Specific aims of this study included developing items for the Weight Literacy Scale; modifying the scale based on survey cognitive testing; and evaluating the scale for reliability and validity.

## Materials and methods

This two-phase study was informed by the six steps of survey development recommended by Hinkin [14]. Phase 1 included inductive item generation with expert content validity assessment. Phase 2 included scale administration to a large sample; exploratory factor analysis and item reduction; assessment of the scale’s internal consistency and validity; and evaluation of the scale’s test re-test reliability. Study procedures for Phases 1 and 2 were approved by the Institutional Review Board of the University of Massachusetts Medical School; all participants provided written informed consent to participate in the study.

### Phase 1

#### Scale development

We first conducted a review of the literature related to health literacy and weight management to operationalize weight literacy (described above). The study team developed preliminary survey items on energy balance and related factors according to key dimensions of weight literacy, including knowledge and awareness of managing energy balance, as well as biological, psychological, behavioral, and environmental (social and physical) factors associated with managing weight. To inform the next iteration of the scale, one-hour, semi-structured interviews were conducted with content experts (N = 9), including a clinical psychologist, an epidemiologist, a dietician, project managers of obesity-related research, and medical students. Survey items were modified based on feedback from the content experts and translated into Spanish by bilingual study team members (a graduate research assistant and the study PI) with expertise in developing materials appropriate for low-literacy populations.

#### Participants and procedures for survey cognitive testing

Cognitive interviewing, a method of improving survey development [15], involved administering items to participants and asking them to explain their understanding of each item and corresponding response options, as described below. Eligibility criteria for participating in cognitive testing of the preliminary survey included: English and/or Spanish-speaking adults (18 years or older); residency in Worcester, MA; and able to provide informed consent to participate. Exclusion criteria included having health or psychiatric conditions that could impair participation in a 1-hour, audio-recorded interview. Study staff recruited participants through outreach to community organizations via in-person, telephone, and email communications. A convenience sample of English-speaking (N = 10) and Spanish-speaking (N = 10) participants were recruited.

Cognitive interviews were conducted by the study team in community locations in Central Massachusetts. During the 1-hour cognitive interviews, participants were asked to read and complete the surveys and highlight confusing or unclear survey items. Study staff then reviewed each survey item with each participant. Participants were asked to paraphrase each item in their own words to identify any problems with comprehension and intended meaning, and study staff noted items that participants perceived as confusing or unclear. All interviews were audio-recorded. Participants received $25 cash for their time to complete the interview. After reviewing the audio-recordings and interview notes, the graduate research assistant and study PI further refined survey items for pilot-testing.

### Phase 2

#### Participants and procedures for survey pilot-testing

Criteria for eligibility for survey pilot-testing (Phase 2) were similar to that of Phase 1: English and/or Spanish-speaking adults (18 years or older); residents of Worcester, MA; and able to provide informed consent to participate. Study staff recruited participants through outreach to community partners and disseminating study information through English- and Spanish-language materials, including flyer postings in community centers and online postings through a clinical research volunteer registry. A total of 200 participants (N = 100 English-speaking and N = 100 Spanish-speaking adults) were recruited for survey pilot-testing. Participants completed a 30-minute in-person visit to complete the Weight Literacy Survey and additional assessments (see Measures section). A subset of participants (N = 71) was invited for a second visit approximately two weeks after the initial visit to complete the survey for test-retest reliability. Assessments took place in a convenient community location or at the medical academic campus. Participants received a $35 gift card for their time.

#### Measures

All Phase 2 measures were collected via in-person assessments at the time of the survey pilot-testing. Participants completed the initial 50-item Weight Literacy Scale, which consisted of true/false and multiple-choice questions. Anthropometric measures (height, weight, and waist circumference measurements) were obtained by trained, bilingual research staff. Weight was measured using portable digital scales (readings were recorded to the nearest 2/10^th^ pound), height was measured to the nearest 1/8^th^ inch using portable stadiometers, and waist circumference was to the nearest 1/8^th^ inch using tape measures. BMI was calculated as weight in kilograms divided by meters squared (kg/m^2^). BMI-based weight status categories included: underweight (<18.5 kg/m^2^), healthy weight (18.5–24.9 kg/m^2^), overweight (25.0–29.9 kg/m^2^), or obese (≥30.0 kg/m^2^).

Dietary intake, physical activity, and sociodemographics were obtained through self-reported, self-administered surveys. Dietary intake was assessed using the Block Rapid Food Screener, a validated measure for identifying high-fat intake and low-fruit/vegetable intake [16]. Physical activity was assessed using the Brief Physical Activity Questionnaire [17], a validated measure of weekly minutes of physical activity. Sociodemographics assessed included: gender, age, race, ethnicity, marital status, education level, annual household income level, current employment status, and country of origin. Participants were also asked to rate their level of confidence in filling out medical forms on their own.

#### Statistical analysis

Frequencies for categorical variables and means and standard deviations for continuous variables were computed to describe Phase 2 (survey pilot-testing) study sample characteristics. Participants’ responses on the initial 50-item Weight Literacy Scale were scored as correct or incorrect for each item, with higher scores indicating greater level of weight literacy. Three rounds of factor analyses were used to determine the final composition of the Weight Literacy Scale. To ensure each item’s unique contribution, variables with factor loadings of .40 or greater were considered to load on that factor[18]. Items that did not achieve factor loadings of .40 or greater were excluded from the scale. Principle component factor analysis was initially forced to 3 factors. Ten questions were eliminated in the first round. As this scale is intended to be a homogeneous measure, the second and third round of factor analysis forced the items to load onto one factor. Nine additional questions were eliminated in the second and third rounds.

Tests of reliability and validity were conducted using the final scale based on results of the factor analyses. Because the item scoring mechanism is binary, we used the Kuder Richardson Coefficient of reliability [19] to determine the scale’s internal consistency. The Kuder Richardson Coefficient of reliability was calculated for the overall study sample and by language (English vs. Spanish). Test-retest reliability was assessed using Pearson’s correlation between average weight literacy scale scores at baseline and 2 weeks follow-up among a subset of Phase 2 participants (N = 71). To examine construct validity, associations between Weight Literacy Scale scores and gender, education level, self-efficacy in filling out medical forms, BMI, weight status, fruit and vegetable intake (servings per day and fruit and vegetable dietary Block scores), and physical activity (minutes of physical activity per week and meeting guidelines of ≥ 150 minutes of physical activity per week) were explored using Pearson Correlation for continuous variables and bivariate regression modeling for categorical variables. Analyses were preformed using Stata version 12 (StataCorp. 2015. *Stata Statistical Software*: *Release 14*. College Station, TX: StataCorp LP).

## Results

### Sample characteristics

Of the 200 participants in Phase 2 of the study, 62.0% were female, the mean age was 41.8 (SD = 14.9), 50.5% reported having less than a college degree, and 58.5% reported an annual household income of less than $40,000. The mean BMI of participants was 30.3 kg/m^2^ (SD = 6.9), with 33.0% of participants falling in the overweight category and 43.5% in the obese category. Over half of participants reported feeling extremely (42.5%) or quite a bit (24.0%) confident in their ability to fill out medical forms on their own, though this varied by primary language spoken (83.0% of English-speaking participants reported feeling ‘extremely’ or ‘quite a bit’ confident vs. 50.0% of Spanish-speaking participants; p<0.001). Additional details of study sample characteristics and differences by primary language spoken are presented in Table 1.

**Table 1:** Study population characteristics, overall and by primary language spoken (N = 200).

		Primary Spoken Language		
	Total (N = 200)	English (N = 100)	Spanish (N = 100)	P-Value*
	N (%)	N (%)	N (%)	
**Gender**				0.37
Male	69 (34.5%)	31 (31%)	38 (38%)	
Female	124 (62%)	65 (65%)	59 (59%)	
Unknown (Missing Data)	7 (3.5%)	4 (4%)	3 (3%)	
**Marital Status**				0.33
Single (never married)	79 (39.5%)	44 (44%)	35 (35%)	
Married/Living with a partner as married	50 (25%)	25 (25%)	25 (25%)	
Separated/Divorced/Widowed	67 (33.5%)	29 (29%)	38 (38%)	
Unknown (Missing Data)	4 (2%)	2 (2%)	2 (2%)	
**Education Level**				<0.001
Elementary school (< 6 years)	10 (5%)	0 (0%)	10 (10%)	
Secondary (6–12 years)	29 (14.5%)	4 (4%)	25 (25%)	
High school diploma	62 (31%)	26 (26%)	36 (36%)	
College	59 (29.5%)	41 (41%)	18 (18%)	
Graduate school	26 (13%)	22 (22%)	4 (4%)	
Unknown (Missing Data)	14 (7%)	7 (7%)	7 (7%)	
**Race**				<0.001
White	80 (40%)	59 (59%)	21 (21%)	
Black or African American	25 (12.5%)	18 (18%)	7 (7%)	
American Indian or Alaskan Native	2 (1%)	0 (0%)	2 (2%)	
Asian	5 (2.5%)	5 (5%)	0 (0%)	
Other	82 (41%)	14 (14%)	68 (68%)	
Unknown (Missing Data)	6 (3%)	4 (4%)	2 (2%)	
**Ethnicity**				<0.001
Hispanic/Latino	87 (43.4%)	15 (15%)	72 (72%)	
Non-Hispanic/Latino	5 (2.5%)	5 (5%)	0 (0%)	
Other	96 (48%)	69 (69%)	27 (27%)	
Unknown (Missing Data)	12 (6%)	11 (11%)	1 (1%)	
**Nativity**				<0.001
US Born	101 (50.5%)	79 (79.0%)	22 (22.0%)	
Non-US Born	98 (49.0%)	42 (21.0%)	77 (77.0%)	
Unknown (Missing Data)	1 (0.5%)	0 (0%)	1 (1.0%)	
**Employment Status**				0.12
Employed	101 (50.5%)	51 (51.0%)	50 (50.0%)	
Unemployed	26 (13.0%)	10 (10.0%)	16 (16.0%)	
Retired	14 (7.0%)	9 (9.0%)	5 (5.0%)	
Homemaker/student	23 (11.5%)	13 (13.0%)	10 (10.0%)	
Disabled	8 (4.0%)	1 (1.0%)	7 (7.0%)	
Unknown (Missing Data)	28 (14.0%)	16 (16.0%)	12 (12.0%)	
**Income**				<0.001
$0-$14,999	60 (30.0%)	25 (25.0%)	35 (35.0%)	
$15,000-$39,999	57 (28.5%)	23 (23.0%)	34 (34.0%)	
$40,000 +	47 (23.5%)	37 (37.0%)	10 (10.0%)	
Don't know/Refused	32 (16.0%)	13 (13.0%)	19 (19.0%)	
Unknown (Missing Data)	4 (2.0%)	2 (2.0%)	2 (2.0%)	
**Body Mass Index (BMI)**				0.38
Healthy weight	46 (23.0%)	27 (27.0%)	19 (19.0%)	
Overweight	66 (33.0%)	33 (33.0%)	33 (33.0%)	
Obese	87 (43.5%)	40 (40.0%)	47 (47.0%)	
Unknown (Missing Data)	1 (0.5%)	0 (0%)	1 (1.0%)	
**Confidence in Filling Out Medical Forms By Yourself**				<0.001
Extremely	85 (42.5%)	57 (57.0%)	28 (28.0%)	
Quite a bit	48 (24.0%)	26 (26.0%)	22 (22.0%)	
Somewhat	41 (20.5%)	14 (14.0%)	27 (27.0%)	
A little bit	13 (6.5%)	3 (3.0%)	10 (10.0%)	
Not at all	12 (6.0%)	0 (0.0%)	12 (12.0%)	
Unknown (Missing Data)	1 (0.5%)	0 (0.0%)	1 (1.0%)	

* P-value obtained from Fisher’s Exact test

### Factor analysis

Of the 50 initial scale items, 31 had factor loadings of 0.40 or greater (see Table 2). Ten items were excluded in round 1, 8 items were excluded in round 2, and 1 item was excluded in round 3 of the factor analysis, resulting in 31 items included in the final scale (see S1 Table). The item difficulty index (percent of respondents who answered each question correctly) for the final scale ranged from 35.5% to 90.0% (see Table 2).

**Table 2:** Distribution of scores and factor loadings on the initial 50-item weight literacy scale (note: items 44 and 45 contain multiple questions; each question counted as an item).

Item	Responses, % Correct	Round 1 Factor Loading	Round 2 Factor Loading
1. People can lose weight without exercising.*	65.5%	0.36	—
2. Drinking water instead of juice can help a person lose weight.	83.0%	0.43	0.45
3. Certain moods can make people want to eat high-calorie foods.	82.5%	0.54	0.61
4. Any physical activity burns calories.	86.0%	0.52	0.62
5. Having friends that are physical active can help a person be more active.	88.5%	0.53	0.47
6. A person can lose weight by eating the same foods, but in smaller portions.*	80.0%	0.41	0.37
7. In equal amounts, fried foods have fewer calories than grilled foods.	73.0%	0.47	0.60
8. Alcoholic beverages have few calories.	72.5%	0.71	0.71
9. Regular meats have fewer calories than lean meats.	58.5%	0.52	0.67
10. The only way to lose weight is eating healthy foods.	49.0%	0.62	0.51
11. Tracking what we eat can help us understand how to cut calories.	86.0%	0.64	0.75
12. People who fast tend to eat more calories.*	48.0%	0.38	—
13. To keep their weight stable, some people need to eat more calories than other people.	65.0%	0.41	0.52
14. Adults who are trying to lose weight should weigh themselves at least once a week.*	65.5%	0.51	0.25
15. In equal amounts, whole milk has fewer calories than 2% milk.*	65.0%	0.32	—
16. Some salad dressings and vinaigrettes can add many calories to a salad.*	79.0%	0.66	0.72
17. In equal amounts, mustard has fewer calories than mayonnaise.	52.0%	0.49	0.62
18. A small glass of orange juice has about the same number of calories as an orange.*	33.5%	0.48	0.36
19. The recommended serving size of cheese for a sandwich is a thin slice.*	57.5%	0.56	0.41
20. One tablespoon of most oils has about 120 calories.*	29.0%	0.63	0.26
21. A brisk 20-minute walk can burn the calories from eating a medium order of French fries.*	29.0%	0.59	0.34
22. A packet (1 teaspoon) of sugar has 40 calories.*	13.0%	0.46	0.22
23. A lunch that has 1,500 calories is healthy for most adults.	52.0%	0.41	0.56
24. An overweight adult who does not exercise needs to eat about 500 fewer calories a day to lose one pound per week.	35.5%	0.51	0.54
25. Exercising one time per day for 30 minutes or exercising three times per day for 10 minutes have the same effect for weight loss.*	37.5%	0.30	—
26. A regular can of non-diet soda has about 10 teaspoons of sugar.*	40.5%	0.42	0.31
27. One teaspoon of sugar has twice the calories of a teaspoon of honey.*	17.5%	0.38	—
28. A cup of any fruit has about 60 calories.*	14.0%	0.55	0.10
29. The recommended serving size of rice (cooked) is one cup.*	16.0%	0.38	—
30. A weight loss goal of 1–2 pounds per week is commonly recommended.	68.5%	0.66	0.55
31. A healthy snack should contain at least 300 calories.	39.0%	0.57	0.49
32. 100% fruit juice contains very few calories.	45.5%	0.59	0.61
33. Healthy snacks can have many calories.*	54.0%	0.36	—
34. A calorie tells us how healthy a food is.	45.5%	0.64	0.62
35. Regular energy drinks contain few calories.	66.5%	0.52	0.59
36. People tend to overeat when there is a lot of food around them.	81.5%	0.69	0.69
37. Setting goals for changing diet and physical activity can help people lose weight.	89.0%	0.66	0.75
38. Eating fried foods less often can help a person lose weight.	66.5%	0.48	0.66
39. Eating smaller portions can help people lose weight.	90.0%	0.71	0.81
40. How many calories a day should an active man eat to have a healthy weight? (An example of an active man is someone who walks briskly for 30 minutes on most days of the week).	57.0%	0.46	0.57
41. How many calories a day should an active woman eat to have a healthy weight? (An example of an active man is someone who walks briskly for 30 minutes on most days of the week).	59.0%	0.53	0.68
42. How many calories a day should an active child aged 5–11 eat to have a healthy weight? (An example of an active child is a girl or boy who plays sports for 60 minutes a day).*	22.0%	0.28	—
43. How many calories a day should an active child aged 12–18 eat to have a healthy weight? (An example of an active adolescent is someone who plays sports for 60 minutes a day).*	12.5%	0.31	—
44a. Based on this pizza label, one serving has 380 calories.	68.0%	0.50	0.46
44b. Based on this pizza label, the entire pizza has 3 servings.	66.5%	0.76	0.75
44c. Based on this pizza label, if you ate the whole pizza, you would be eating 760 calories.	56.5%	0.54	0.69
45a. Based on this soda container label, one serving has 150 calories.	77.0%	0.77	0.65
45b. Based on this soda container label, the entire soda has 2 servings.	59.5%	0.80	0.63
45c. Based on this soda container label, if you drank the entire soda bottle, you would be drinking 300 calories.	63.0%	0.79	0.64
46. Exercising one time per day for 30 minutes burns the same number of calories as exercising three times per day for 10 minutes.*	36.0%	0.33	—

*Item not included in final scale.

### Weight literacy scale

Participants’ mean Weight Literacy Scale score on the final 31-item scale was 20.6 (SD = 7.0). The median score was 21.0; range of 0–31.0. Women on average scored 2 points higher on the scale compared to men (p = 0.05).

### Internal consistency

The overall Kuder Richardson Coefficient of reliability for the final Weight Literacy Scale was 0.90. Similar coefficients were found by language (range of 0.84–0.88).

### Construct validity

Participants’ weight literacy scores were positively associated with education level (β = 3.6; 95% CI: 2.8–4.3); p<0.001) and confidence in ability to fill out medical forms on their own (β = 7.5; 95% CI: 5.8–9.4; p<0.001). Those who reported being ‘extremely’ or ‘quite a bit confident’ in filling out medical forms on their own on average scored 7.5 points higher on the Weight Literacy Scale than those who reported feeling ‘somewhat,’ ‘a little bit,’ or ‘not at all confident’ in filling out medical forms. Weight Literacy Scale scores were not significantly correlated with minutes of physical activity per week, number of fruit and vegetable servings, fruit and vegetable dietary Block score, or BMI. Weight Literacy Scale scores were not significantly associated with meeting physical activity guidelines (≥ 150 minutes of physical activity per week) or weight status category.

### Test-retest reliability

The Pearson’s correlation coefficient between the final Weight Literacy Scale scores at baseline and at 2-week follow-up among the subset of Phase 2 participants (N = 71) was 0.72 (95% CI: 0.60–0.82).

## Discussion

This two-phase study describes the development of the Weight Literacy Scale, an instrument designed to measure the degree of individuals’ capability to obtain, process, and understand weight loss information needed to make informed decisions regarding weight management, and the results of psychometric tests of the scale among 200 English- and Spanish-speaking adults.

### Principal findings

Study findings support the reliability and validity of using the Weight Literacy Scale to assess weight literacy among a predominantly low socioeconomic status, ethnically diverse population. Weight Literacy Scale scores did not correlate with participants’ diet, physical activity, or BMI, but were significantly associated with gender and positively associated with education level.

### Interpretation of findings

On average, participants answered two thirds of the Weight Literacy Scale items correctly (20.6 out of 31 items), indicating substantial room for improvement in weight literacy.

As expected, Weight Literacy Scale scores were positively associated with education level and self-efficacy to complete medical forms. Participants who reported a higher degree of confidence in being able to complete medical forms on their own had on average higher Weight Literacy Scale scores than those who reported less confidence. Weight Literacy Scale scores were not significantly associated with measures of diet, physical activity, BMI, or weight status. A possible explanation for this finding is that while individual-level literacy in weight control may facilitate successful weight management, factors at multiple levels (individual, interpersonal, community, and environmental) across multiple domains shape individuals’ diet, physical activity, and weight. For example, individuals with high weight literacy may still face substantial environmental and social barriers to achieving healthy eating and physical activity goals needed for weight loss [20]. Thus, BMI, weight, and weight management behaviors (e.g., diet, physical activity) may not necessarily be the strongest constructs to validate weight literacy. Previous efforts and experiences to lose weight may be more closely linked with weight literacy, though we did not collect this type of data for this study.

### Clinical implications

Clinical settings and situations where this scale may be useful include interactions with providers and clinicians where weight loss or weight management is discussed as a health goal. The Weight Literacy Scale may serve as a tool to screen individuals who may need more assistance with developing a knowledge base around weight loss and with making informed decisions as part of weight loss or weight management (e.g., healthy gestational weight gain among pregnant women) interventions. Individuals’ responses on the scale can inform which strategies are needed to promote comprehension, self-efficacy, and/or skills needed for weight loss among those with low weight literacy. Though this scale was tested among individuals with lower educational levels (>70% had a high school degree or less), the scale may be administered to adult populations with varying levels of education, including college degree or higher.

### Research implications

Overall, the Weight Literacy Scale demonstrates strong internal consistency and reasonable construct validity and test-retest reliability. This study has several strengths, including the development of a novel, linguistically-appropriate weight literacy instrument in English and in Spanish using formative, mixed methods research with an ethnically diverse study sample and conducting test-retest reliability with a subset of the study sample. Though the scale was developed for a broad adult population and not intended solely for individuals seeking weight management, the use of a convenience sample recruited from selected community locations within a city in central Massachusetts limits the generalizability of study findings. Latinos were primarily of Caribbean origin, thus it is unknown whether the instrument would have the same psychometric characteristics in other Hispanic sub-groups (e.g., Mexican Americans). Future research should examine the reliability and validity of the Weight Literacy Scale among other populations and assess participants’ previous efforts to lose weight as a construct for additional validity testing. Findings from this study indicate that the Weight Literacy Scale may be particularly useful as a tool to identify specific target areas for improving individuals’ understanding and capabilities related to weight management in future randomized controlled or quasi-experimental trials or to examine the association between weight literacy and weight-related outcomes in future observational studies.

### Conclusions

Studies on the role of health literacy in the context of behavioral weight management are limited. The Weight Literacy Scale may be used to assess weight literacy in studies that seek to further elucidate the role of this construct in weight loss and management, screen individuals who may need more assistance with making informed decisions related to weight loss and management, as well as help identify educational targets in the context of weight loss interventions.

## Supporting information

S1 Table Final 31-item Weight Literacy Scale–English. Note: Items 26 and 27 contain multiple questions; each question is counted as an item. * denotes the correct answer.
(DOCX)

S1 Dataset Study sample demographics and construct validity data. (CSV)

S2 Dataset Study sample weight literacy score data. (CSV)
