# Supplementary material for: Development and validation of a Weight Literacy Scale in English and Spanish
Source: PLoS One. 2018 Oct 24;13(10):e0204678. doi: 10.1371/journal.pone.0204678 (PMC6200187; doi:10.1371/journal.pone.0204678)
Supplement: S1 Table — Note: Items 26 and 27 contain multiple questions; each question is counted as an item. * denotes the correct answer. (DOCX) [file pone.0204678.s001.docx]

**S1 Table. Final 31-item Weight Literacy Scale – English**

| **Survey #** | **Statement**  *Please read each statement and circle whether it is: True, False or Don’t Know.* |  |  |  |
| --- | --- | --- | --- | --- |
| 1 | Drinking water instead of juice can help a person lose weight. | **True*** | **False** | **Don’t know** |
| 2 | Certain moods can make people want to eat high-calorie foods. | **True*** | **False** | **Don’t know** |
| 3 | Any physical activity burns calories. | **True*** | **False** | **Don’t know** |
| 4 | Having friends that are physically active can help a person be more active. | **True*** | **False** | **Don’t know** |
| 5 | In equal amounts, fried foods have fewer calories than grilled foods. | **True** | **False*** | **Don’t know** |
| 6 | Alcoholic beverages have few calories. | **True** | **False*** | **Don’t know** |
| 7 | Regular meats have fewer calories than lean meats. | **True** | **False*** | **Don’t know** |
| 8 | The only way to lose weight is eating healthy foods. | **True** | **False*** | **Don’t know** |
| 9 | Tracking what we eat can help us understand how to cut calories. | **True*** | **False** | **Don’t know** |
| 10 | To keep their weight stable, some people need to eat more calories than other people. | **True*** | **False** | **Don’t know** |
| 11 | Some salad dressings and vinaigrettes can add many calories to a salad. | **True*** | **False** | **Don’t know** |
| 12 | In equal amounts, mustard has fewer calories than mayonnaise. | **True*** | **False** | **Don’t know** |
| 13 | A lunch that has 1,500 calories is healthy for most adults. | **True** | **False*** | **Don’t know** |
| 14 | An overweight adult who does not exercise needs to eat about 500 fewer calories a day to lose one pound per week. | **True*** | **False** | **Don’t know** |
| 15 | A weight loss goal of 1-2 pounds per week is commonly recommended. | **True*** | **False** | **Don’t know** |
| 16 | A healthy snack should contain at least 300 calories. | **True** | **False*** | **Don’t know** |
| 17 | 100% fruit juice contains very few calories. | **True** | **False*** | **Don’t know** |
| 18 | A calorie tells us how healthy a food is. | **True** | **False*** | **Don’t know** |
| 19 | Regular energy drinks contain few calories. | **True** | **False*** | **Don’t know** |
| 20 | People tend to overeat when there is a lot of food around them. | **True*** | **False** | **Don’t know** |
| 21 | Setting goals for changing diet and physical activity can help people lose weight. | **True*** | **False** | **Don’t know** |
| 22 | Eating fried foods less often can help a person lose weight. | **True*** | **False** | **Don’t know** |
| 23 | Eating smaller portions can help people lose weight. | **True*** | **False** | **Don’t know** |

*For the following four multiple-choice questions, please circle one answer.*

| 24 | How many calories a day should an active man eat to have a healthy weight? (An example of an active man is someone who walks briskly for 30 minutes on most days of the week)   1. Less than 1,000 calories 2. 1,500 to 2,500 calories* 3. 3,000 to 4,000 calories 4. 4,500 calories or more 5. Don’t know |
| --- | --- |
| 25 | How many calories a day should an active woman eat to have a healthy weight? (An example of an active woman is someone who walks briskly for 30 minutes on most days of the week)   1. Less than 1,000 calories 2. 1,500 to 2,500 calories* 3. 3,000 to 4,000 calories 4. 4,500 calories or more 5. Don’t know |

*Please read each nutrition label before answering the questions.*

|  | **This nutrition label is from a pizza box:**  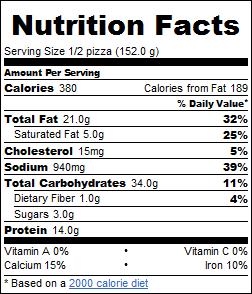  *Based on this pizza label, please circle whether the following three statements are: True, False, or Don’t Know.* | | | |
| --- | --- | --- | --- | --- |
| 26a | One serving has 380 calories. | **True*** | **False** | **Don’t know** |
| 26b | The entire pizza has 3 servings. | **True** | **False*** | **Don’t know** |
| 26c | If you ate the whole pizza, you would be eating 760 calories. | **True*** | **False** | **Don’t know** |
|  | **This nutrition label is from a soda container:**  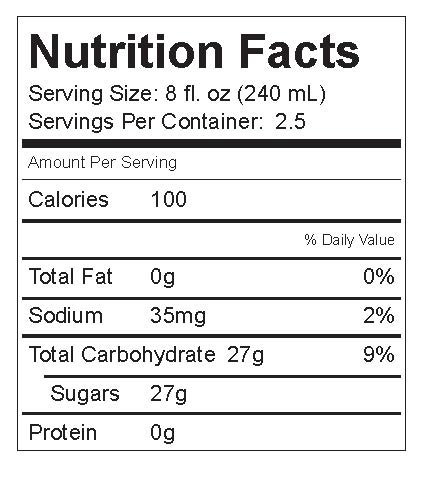  *Based on this soda container label, please circle the following three statements are: True, False, or Don’t Know.* | | | |
| 27a | One serving has 150 calories. | **True** | **False*** | **Don’t know** |
| 27b | The entire soda can has 2 servings. | **True** | **False*** | **Don’t know** |
| 27c | If you drank the entire soda bottle, you would be drinking 300 calories. | **True** | **False*** | **Don’t know** |
